# Complete shutdown of microvascular perfusion upon hepatic cryothermia is critically dependent on local tissue temperature

**DOI:** 10.1054/bjoc.1999.1001

**Published:** 2000-02

**Authors:** G Schüder, G Pistorius, M Fehringer, G Feifel, M D Menger, B Vollmar

**Affiliations:** Department of General Surgery and Institute for Clinical and Experimental Surgery, University of Saarland, Homburg/Saar, 66421, Germany

**Keywords:** cryosurgery, liver microcirculation, tissue temperature, sinusoidal perfusion failure

## Abstract

Since microvascular dysfunction with complete circulatory arrest and, thus, prolongation of tissue ischaemia is considered a potential mechanism for cell necrosis following hepatic cryosurgery, we determined the temperature necessary for induction of complete nutritive perfusion failure in cryothermia-treated rat livers. After localization of the cryoprobe with seven thermocouples and application of a single or double freeze–thaw cycle, in vivo fluorescence microscopy of the cryoinjured left lobe was performed over a 2-h period using a computer-controlled stepping motor, which guaranteed analysis of the identical liver tissue segments with exact allocation of the thermocouples and thus determination of tissue temperature. Cryothermia resulted in a central non-perfused part of injury, surrounded by a heterogeneously perfused peripheral zone. The non-perfused area after single and double freezing continuously increased over the first 90-min period due to a successive shutdown of perfusion within the peripheral border zone. Analysis of the thermocouples' temperature at the end of freezing revealed the 0°C-front at 11.7 mm (single freeze–thaw cycle) and 12.1 mm (double freeze–thaw cycle) distant from the centre of the cryoprobe, which exactly corresponds with the initial (30 min) expansion of the area with nutritive perfusion failure. The increased non-perfused tissue area at 2 h conformed a critical border temperature between 8.29 ± 1.63°C and 9.07 ± 0.24°C. From these findings, we conclude that freezing of liver tissue to temperatures of at least < 0°C causes complete/irreversible perfusion failure, which consequently will result in cell death and tissue necrosis, and may thus be supposed as a prerequisite for the safe and successful application of cryosurgery in hepatic tumour ablation. © 2000 Cancer Research Campaign


British Journal of Cancer (2000) **82**(4), 794–799
© 2000 Cancer Research Campaign
DOI: 10.1054/ bjoc.1999.1001, available online at http://www.idealibrary.com on IDEAL®

### Complete shutdown of microvascular perfusion upon hepatic cryothermia is critically dependent on local tissue temperature

**G Schüder[1], G Pistorius[1], M Fehringer[1], G Feifel[1], MD Menger[2] and B Vollmar[2]**

[1]Department of General Surgery and [2]Institute for Clinical and Experimental Surgery, University of Saarland, 66421 Homburg/Saar, Germany

**Summary** Since microvascular dysfunction with complete circulatory arrest and, thus, prolongation of tissue ischaemia is considered a potential mechanism for cell necrosis following hepatic cryosurgery, we determined the temperature necessary for induction of complete nutritive perfusion failure in cryothermia-treated rat livers. After localization of the cryoprobe with seven thermocouples and application of a single or double freeze–thaw cycle, in vivo fluorescence microscopy of the cryoinjured left lobe was performed over a 2-h period using a computer-controlled stepping motor, which guaranteed analysis of the identical liver tissue segments with exact allocation of the thermocouples and thus determination of tissue temperature. Cryothermia resulted in a central non-perfused part of injury, surrounded by a heterogeneously perfused peripheral zone. The non-perfused area after single and double freezing continuously increased over the first 90-min period due to a successive shutdown of perfusion within the peripheral border zone. Analysis of the thermocouples' temperature at the end of freezing revealed the 0°C-front at 11.7 mm (single freeze–thaw cycle) and 12.1 mm (double freeze–thaw cycle) distant from the centre of the cryoprobe, which exactly corresponds with the initial (30 min) expansion of the area with nutritive perfusion failure. The increased non-perfused tissue area at 2 h conformed a critical border temperature between 8.29 ± 1.63°C and 9.07 ± 0.24°C. From these findings, we conclude that freezing of liver tissue to temperatures of at least < 0°C causes complete/irreversible perfusion failure, which consequently will result in cell death and tissue necrosis, and may thus be supposed as a prerequisite for the safe and successful application of cryosurgery in hepatic tumour ablation. © 2000 Cancer Research Campaign

**Keywords:** cryosurgery; liver microcirculation; tissue temperature; sinusoidal perfusion failure

Cryothermia is the use of freezing to induce tissue destruction. In the treatment of both metastatic colorectal and non-colorectal tumours as well as primary hepatic tumours, cryogenic therapy has become an attractive alternative to surgical resection in patients whose underlying medical condition is thought to preclude resection due to limited hepatic reserve and those with lesions either involving multiple lobes or being anatomically unsuitable for resection (Dalton and Eisenberg, 1993; Morris et al, 1993; Cozzi et al, 1995; Adam et al, 1997; Lee et al, 1997). Moreover, cryosurgery is ideally suited for patients with recurrent hepatic disease because of limited destruction of normal tissue. Beside the radiologic disappearance of lesions, follow-up of serum tumour markers (carcinoembryonic antigen (CEA), CA-125, 5-hydroxy-indoleacetic acid (HIAA), gastrin, serotonin, vasoactive intestinal polypeptide (VIP), cortisol, glucagon, alpha-fetoprotein (AFP), CA 15-3, CA 19-9) has shown the decrease in the serial tumour marker levels, giving some evidence of therapeutic efficacy (Ravikumar et al, 1987; Charnley et al, 1989; Ravikumar and Steele, 1989; Ravikumar et al, 1991; Cozzi et al, 1995; Bilchik et al, 1997; Hamad and Neifeld, 1998). However, a significant problem limiting the use of this technology is the high rate of local recurrence at the cryosite after technically successful cryosurgical tumour ablation (Ravikumar et al, 1991; Johnson et al, 1997). Recent results indicate inadequate tumour ablation in a third of patients who were thought to have been treated adequately, supposedly because of insufficient temperatures at the tumour edge when treating large lesions (Seifert and Morris, 1999).

Detailed studies of the response of human tumour and normal liver to freezing as well as studies of the response of human tumour cell lines in animals to cryothermia have been reported (Jacob et al, 1985; Ravikumar et al, 1991). Intracellular ice crystal formation is suggested to be the predominant cause for cryothermia-induced tumour necrosis. In addition, denaturation of membrane lipid protein complexes, osmotic changes during thawing and local tissue ischaemia as a result of vascular thrombosis are proposed as further mechanisms responsible for tumour destruction by freezing (Gage, 1969; Smith et al, 1978; Whittaker, 1984; Rubinsky et al, 1990). Although of particular importance for induction of cell necrosis and tissue damage, microcirculatory aspects, i.e. impairment of nutritive organ blood flow with inadequate oxygen delivery, have only gained minor interest in the research fields of cryothermia.

In previous studies, Brown and co-workers have demonstrated reduced blood flow in normal liver and liver tumour tissue over the following 8 h upon cryosurgery (Brown et al, 1993) and complete microvascular shutdown in a cremaster muscle preparation within 2 h after cryotherapy (Brown et al, 1994), strongly indicating the contribution of microcirculatory dysfunction to tissue/tumour necrosis in situ. Further, it had been shown that in vivo freezing causes cytotoxic cell damage which, however, is not lethal to all tumour cells, but requires microvascular injury with

*Received 10 May 1999*
*Accepted 14 September 1999*

*Correspondence to:* B Vollmar subsequent perfusion failure and deprivation of oxygen to prevent tumour recurrence (Bayjoo, 1992). The freezing temperature necessary to irreversibly induce shutdown of microvascular perfusion in liver tissue, however, has not been determined yet. Thus, the aim of this study was to determine the critical freezing temperature necessary to induce complete shutdown of microvascular/sinusoidal perfusion of hepatic tissue in vivo using a fluorescent microscopic approach.

### MATERIALS AND METHODS

### Animals and surgical procedure

In accordance with the German legislation on protection of animals and the 'Guide for the care and use of laboratory animals' (NIH publication no. 86-23, revised 1985), experiments were performed in ten 16- to 20-week-old Sprague-Dawley rats with a body weight of 300–400 g. Following anaesthesia by intraperitoneal (i.p.) injection of pentobarbital (50 mg $kg^{-1}$ body weight), the animals were placed on an electronically regulated heating pad, which continuously adjusted the body temperature to 37°C. Tracheotomy was performed to facilitate spontaneous breathing. The right carotid artery and jugular vein were catheterized for continuous macrohaemodynamic monitoring and application of sodium-fluorescein for intravital microscopy.

After median laparotomy, the rats were positioned on their left side and the livers were prepared for intravital fluorescence microscopy by placing the left lobe on a plasticine disk held by an adjustable stage that was attached to the heating pad. Thereby, the lower surface of the liver was situated horizontal to the microscope, which guaranteed an adequate focus level for the microscopic procedure on the area of liver surface under investigation. In addition, the adjustment of the plasticine disk allowed avoidance of mechanical obstruction of feeding and draining macrovessels and minimized respiratory movements of the lobe mediated by the diaphragma. The exposed area of the left liver lobe was immediately covered with a glass slide to prevent drying of tissue and the influence of ambient oxygen (Vollmar et al, 1994, 1997). The animals were then allowed a stabilization period of approximately 30 min before induction of cryothermia.

### Intravital fluorescence microscopy

The animals on the heating pad were transferred to the stage of a Zeiss Axio-Tech microscope (Zeiss, Oberkochen, Germany) equipped with a mercury arc lamp (100 W) for epi-illumination fluorescent light microscopy. Microscopic images were registered by a charge-coupled device video camera (FK 6990; Prospective Measurements Inc., San Diego, CA, USA) and were transferred to a video system (VO-5800 PS; Sony GmbH, Munich, Germany). With the use of a long distance ×10 objective (10×/0.30, Zeiss) and a water immersion objective (W 20×/0.5, Zeiss) magnifications of ×300 and ×600 were achieved on the video screen (PVM-1442 QM, diagonal: 330 mm, Sony). By means of blue epi-illumination (450–490 nm excitation wavelength/>520 nm emission wavelength), microfluorographic images of the hepatic surface were taken after intravenous (i.v.) injection of sodium-fluorescein which allows in vivo contrast enhancement for improved visualization of the hepatic microvasculature.

### Cryothermia

Cryothermia was induced to the left liver lobe of ten animals using a 6-mm-diameter cylindrical cryoprobe (Erbokryo PS; Erbe, Tübingen, Germany), cooled to –160°C by internally circulating liquid nitrogen. For freezing, the cryoprobe was placed carefully upon the surface of the left liver lobe together with seven thermocouples (TC1–TC7) in a final distance of 5, 7, 9, 11, 13, 15 and 17 mm from the centre of the cryoprobe. Exact localization of the thermocouples was guaranteed by a plexiglas holder mounted to the cryoprobe and confirmed by in vivo microscopy using the computer-controlled stepping motor. The propagation of the macroscopically visible ice-front allowed gross assessment of the tissue area involved during the 3-min period of freezing, which was required to achieve a tissue temperature of –60°C at TC1. Temperatures of the seven thermocouples were assessed in 30-s intervals during both the time of freezing and the following 17 min of thawing. At the end of the freezing procedure, tissue temperature measurements revealed values below –60°C in the central portion of the cryolesion (TC1). In five animals the hepatic microcirculation was studied after one freeze–thaw cycle over a 2-h period using intravital microscopy. In five further animals the freezing procedure was repeated a second time for 3 min after having allowed a 2-min intermittent thawing period, which then was followed by the intravital microscopic studies during 2 h after thawing.

### Experimental protocol and microcirculatory analysis

Time points of microcirculatory studies were 30, 45, 60, 75, 90, 105 and 120 min after termination of freezing. In each animal, sodium fluorescein for in vivo contrast enhancement of the hepatic microcirculation was injected 5 min prior to the first time point of microcirculatory analysis. Using a computer-controlled stepping motor, the identical hepatic tissue region around the cryoprobe was repetitively analysed in terms of (i) sinusoidal perfusion failure, i.e. complete cessation of perfusion within all sinusoids visible, (ii) heterogeneous perfusion, as well as (iii) regular sinusoidal perfusion, i.e. sinusoidal perfusion rate >98%. Hepatic tissue zones exhibiting perfusion failure or heterogeneous perfusion are given in radial length with respect to the centre of the cryoprobe. After the 120-min observation period, animals were sacrificed and liver tissue was removed for fixation in 10% buffered formaldehyde solution and subsequent haematoxylin and eosin staining for light microscopy.

### Statistical analysis

Data are given as mean ± s.e.m. To test for differences between groups, a Kruskal–Wallis one-way analysis of variance on ranks (overall differences) was performed, followed by the appropriate all pairwise multiple comparison procedure (Dunn's method). To test for differences between repeated measurements at different time points, Friedman repeated measures analysis of variance on ranks was used. To assess the relation between the thermocouples' location and their temperature at the end of freezing, regression analysis was performed. Calculation of the coefficient of variance (standard deviation/mean) served as a measure of heterogeneity. Statistics were performed using the software package SigmaStat (Jandel Corporation, San Rafael, CA, USA). A $P$-value of $< 0.05$ was considered statistically significant.

*© 2000 Cancer Research Campaign*

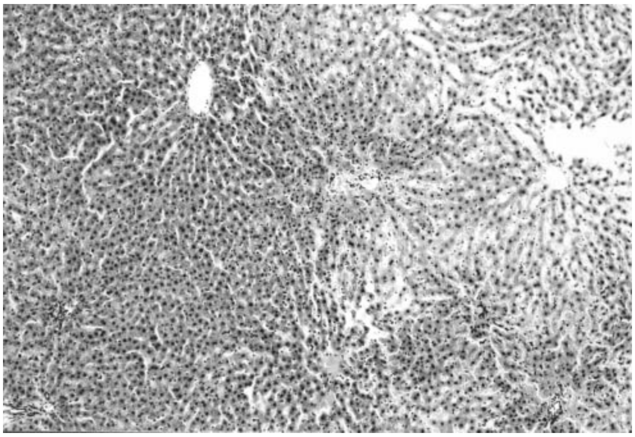

**Figure 1** Haematoxylin-eosin stained section of a rat left liver lobe after cryothermia to – 60°C for 3 min. Note the coagulative necrosis throughout the cryolesion with pyknosis and loss of cell boundaries, which clearly demarcates from the normal hepatic tissue. No histological damage was found outside the cryolesion. Magnification ×120

### RESULTS

The procedure of cryothermia was tolerated by all animals without major haemorrhage or cracking of the liver surface upon freezing. At the end of the 3-min freezing period and subsequent passive thawing of the liver tissue, gross examination of the liver surface revealed a clear border between non-injured normal tissue and the cryolesion (Figure 1). Concomitantly, in both groups, histological evaluation demonstrated complete coagulative necrosis with a clear demarcation between the cryolesion and the normal hepatic tissue, which did not differ when compared with the histological appearance of an untreated rat liver (Figure 1).

Using the centre of the cryoprobe as starting point, computer-controlled repetitive scanning of identical tissue regions within each animal allowed us to study the expansion of the cryolesion over time with assessment of dynamics of tissue perfusion within both the centre and the bordering zone of the cryolesion (Figure 2). Quantitative analysis revealed a non-perfused central area with a radial length of 11.61 ± 0.75 mm after application of one freeze–thaw cycle, which gradually increased up to a length of 13.38 ± 0.70 mm at the end of the 2-h observation period without any recovery of nutritive perfusion (Figure 3). The increase of area of completely non-perfused tissue was due to a successive decrease of the size of the heterogeneously perfused surrounding border zone. The radial length of this border zone was initially 1.28 ± 0.43 mm (30 min), but was found reduced to 0.66 ± 0.48 mm at 2 h after freezing due to progressive microvascular shut-down of perfusion. Comparable results were obtained in animals which underwent two subsequent freeze–thaw cycles, except the fact that the size of the heterogeneously perfused border zone was found somewhat smaller (30 min: 0.71 ± 0.12 mm; 2 h: 0.38 ± 0.25 mm) in favour to a slightly, but not significantly, increased size of non-perfused tissue (Figure 3). Comparison between the two groups concerning the coefficient of variance (relative dispersion) for the radial length of the non-perfused and heterogeneously perfused areas over the 2-h observation period, however, revealed markedly lower values in the group with two freezing cycles (range 0.020–0.082 and 0.338–1.471), when compared with those of the single freezing procedure (range 0.114–0.145 and 0.751–1.63), indicating a more homogeneous manifestation of

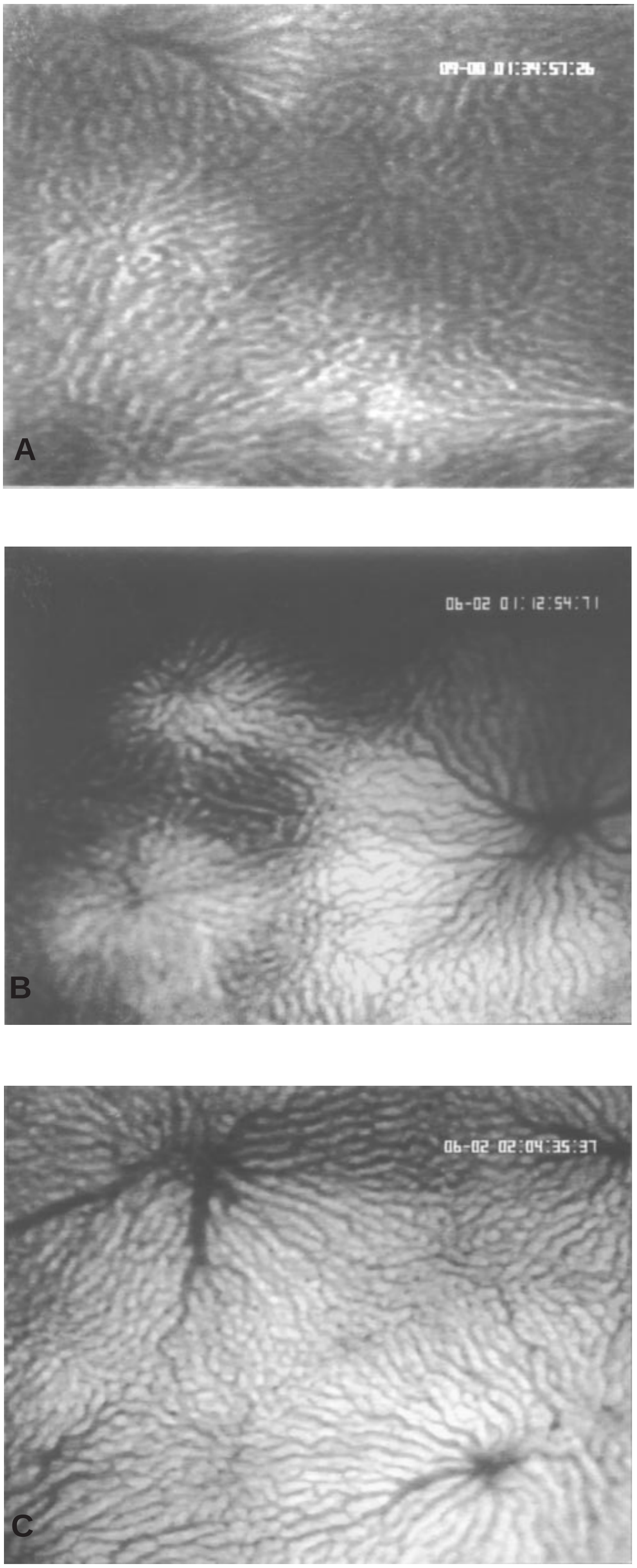

**Figure 2** Intravital fluorescence microscopy of rat liver microcirculation after cryothermia visualized by blue light epi-illumination and sodium-fluorescein contrast enhancement. Note complete sinusoidal perfusion failure within the central zone of the cryolesion **(A)** and the heterogeneously critically perfused border zone in the periphery **(B)**. Hepatic microcirculation within the tissue area which is in direct vicinity to the cryolesion appears normal **(C)**. Original magnification ×110

© 2000 Cancer Research Campaign

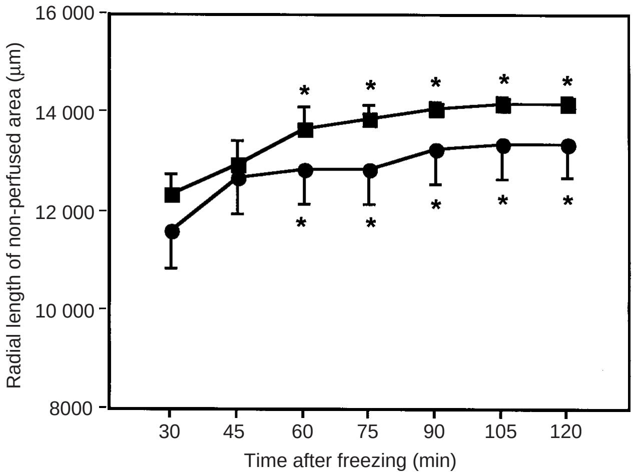

**Figure 3** Radial length of the non-perfused tissue area of the cryolesion after application of a single (circles, *n* = 5) and a double (squares, *n* = 5) freeze–thaw cycle to the left liver lobe of rats. Analysis of sinusoidal perfusion failure was performed using intravital fluorescence microscopy and blue epi-illumination after intravenous sodium-fluorescein contrast enhancement. By means of a computer-controlled stepping motor, identical tissue regions within each animal were analysed in terms of expansion during the 30- to 120-min observation period. Means ± s.e.m.; *$P < 0.05$ vs 30-min value.

microvascular injury, probably due to the repetition of the cryo-intervention.

Analysis of the time–temperature profiles of the thermocouples placed in 5–17 mm distances from the centre of the cryoprobe demonstrated the different steepness in the fall of temperature in dependency to the distance between the thermocouple and the cryoprobe (Figure 4). Temperature distribution pattern and changes over time, however, were found similar regardless of whether the hepatic tissue underwent one or two freeze–thaw cycles (Figure 4). Regression analysis revealed a significant ($P < 0.01$) correlation between the site of thermocouples in respect to the cryoprobe and the temperature at the end of the freezing period with regression coefficients of $r^2 = 0.997$ and $r^2 = 0.993$ for the application of the single and the double freeze–thaw cycle (Figure 5). By use of the equations that mathematically represent the relation between tissue temperature and distance to the cryoprobe after the single or double freeze–thaw cycle (Figure 5), the 0°C-front revealed an expansion of 11.66 mm and 12.11 mm after single and double freeze–thaw cycles, which almost exactly correspond with the radial expansion of the mean value of the non-perfused tissue area at 30 min after freezing (11.61 ± 0.75 mm and 12.34 ± 0.42 mm, Figure 3). The subsequent expansion of the non-perfused area with a mean value of 13.38 ± 0.70 mm and 14.16 ± 0.16 mm at 2 h after termination of freezing allowed to deduce mean temperatures of +8.29 ± 1.63°C and +9.07 ± 0.24°C, and to suppose these temperatures critical to induce complete shut-down of microvascular perfusion.

### DISCUSSION

To our knowledge this is the first study quantitatively reporting on the in vivo effect of cryothermia in terms of dimension of hepatic cryolesions and the tissue temperature necessary to cause complete microvascular/sinusoidal perfusion failure at 2 h. With the use of intravital fluorescence microscopy, we were able to assess the dynamics of microvascular response occurring in distinct tissue regions in relation to the tissue temperature at the end of the freezing cycle. We demonstrate that tissue freezing to temperatures of at least less than 0°C is associated with complete shutdown of microvascular perfusion, and might, therefore, be a prerequisite for successful application of cryothermia in liver tumour therapy.

Despite the fact that studies of isolated cell suspensions have identified some of the factors causative for cell death (Jacob et al, 1985; Tatsutani et al, 1996), the response to cold of isolated cell suspensions seems to differ considerably from intact tissue and solid organs. Freezing of cell suspensions underestimates the degree of necrosis in frozen tissue, largely because of the lack of secondary effects of freezing, in particular the lack of microcirculatory arrest and tissue ischaemia (Neel et al, 1971; Raab et al, 1974; Whittaker, 1984). The significance of these host-associated factors is exclusively evident by the present data on the consecutive shutdown of sinusoidal perfusion within the border zone of the cryolesion. This critically perfused rim of the cryolesion is the most likely area for incomplete tissue death and, in case of malignant tissue and tumour growth, the potential source for local recurrence. Given our results, it must be ensured that hepatic cryolesions include a rim of normal liver well beyond the tumour, where at the end of freezing tissue temperatures of 0°C and less are reached. Thus, the present study now strengthens the empirically based design for cryosurgery of liver tumours/metastasis which postulates for achievement of an ‘ice-ball’, exceeding 1 cm the diameter of the lesion (Adam et al, 1997). As now demonstrated in the present study using the integrative approach of in vivo microscopy and temperature monitoring within identical liver tissue regions, we demonstrated that these temperatures cause complete microvascular perfusion failure. Based on chronic experiments, which show complete loss of initially cryoinjured tissue area at 8 weeks after cryotherapy (Schüder et al, 1999), we like to postulate that the microvascular perfusion failure is irreversible in nature without any return of nutritive blood flow. This view explains the observation of Bayjoo (1992) that cryoinjured liver tumours, although still containing viable tumour cells directly after cryothermia, lack tumour regrowth over weeks, because of the shutdown of the microcirculation results in complete ischaemia with failure of nutritive supply of potentially still viable tumour cells.

The observation that double freezing causes a slight increase of the area/volume of frozen tissue confirms and extends previous studies (Gill et al, 1968) in that repetitive freezing increased the homogeneity of the extent of tissue presenting with shutdown of microvascular perfusion, as reflected by the minor dispersion of single values and thus lower coefficients of variance. A homogeneous response of the solid organ to cold would increase the certainty to establish an adequate margin within that cells become irreversibly destructed due to direct freezing effects and/or perfusion failure-associated tissue hypoxia/anoxia. This aspect is again of particular importance for successful cryosurgery of liver tumours, because still viable tumour cells might spread and engraft in the liver or in extra-hepatic organs. Thus, the most common clinical use of two freeze–thaw cycles in hepatic cryosurgery (Ravikumar and Steele, 1989; Zhou et al, 1993; Steele, 1994; Adam et al, 1997) should be continued, despite the fact that along with our histological results experimental studies on hepatic cryoablation in a porcine model did not report on differences between tissue receiving one or two freeze–thaw cycles (Weber

*© 2000 Cancer Research Campaign* 

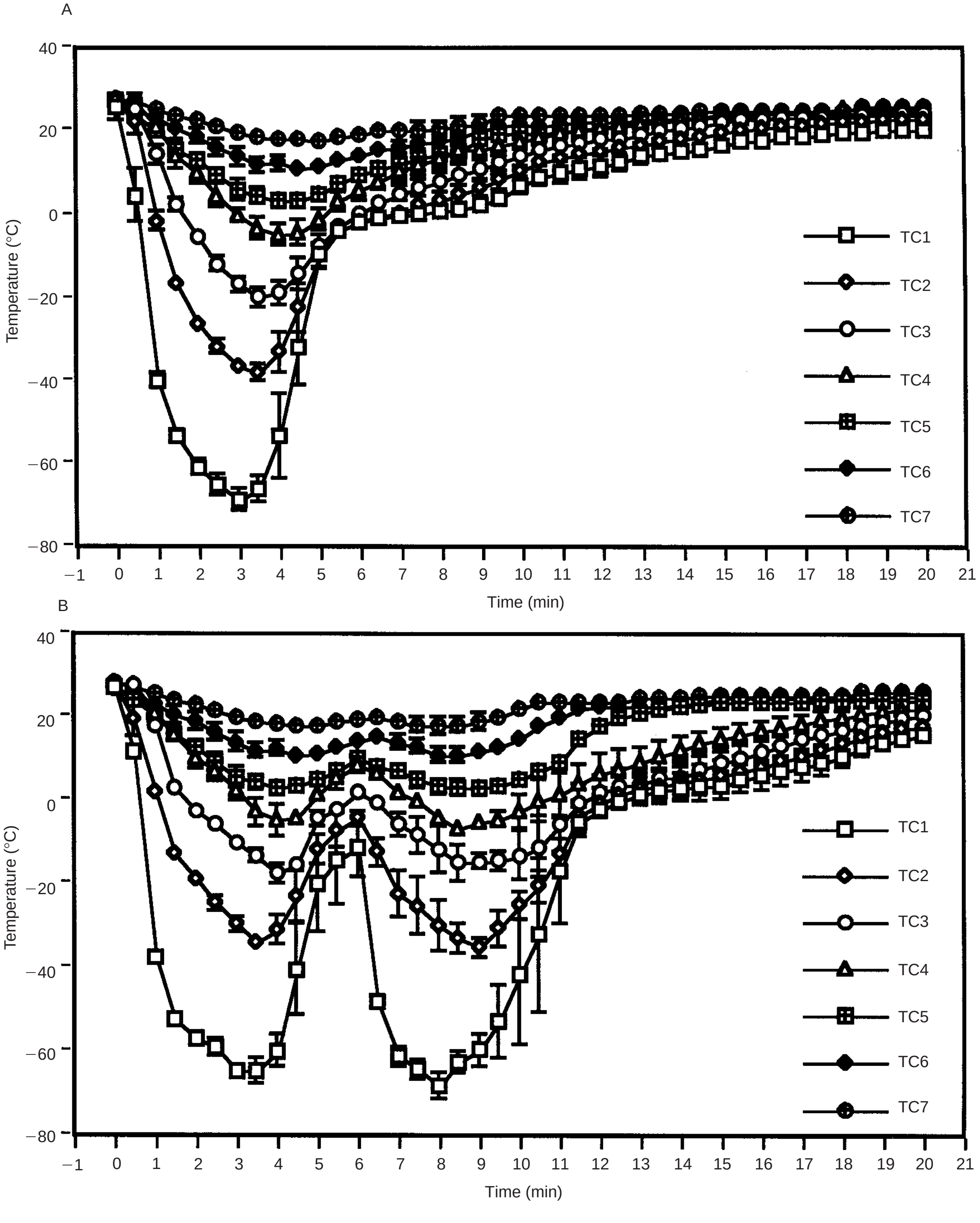

**Figure 4** Time-temperature profiles of seven thermocouples (TC1–TC7) placed in 5-, 7-, 9-, 11-, 13-, 15- and 17-mm distances to the centre of the cryoprobe. Cryolesions were applied to the left liver lobe of rats by either a single **(A)** freezing procedure to –60°C, followed by passive thawing ($n$ = 5) or a double **(B)** freezing procedure to –60°C for 3 min each having allowed a 2-min intermittent thawing period ($n$ = 5). The closer the thermocouples to the cryoprobe are, the steeper is the fall of tissue temperature upon freezing. Means ± s.e.m.

et al, 1997).

In summary, this study has demonstrated in vivo the temperature profile of hepatic cryolesions thereby differentiating between areas of initial complete perfusion failure and heterogeneously perfused border zones, exhibiting subsequently complete microcirculatory shutdown. Since these margins of cryolesions are critical in terms of tumour cell survival and might inevitably predict local recurrence, we deduce from the present results that temperatures below 0°C in normal liver tissue sufficiently distant to the border of the tumour mass should be a prerequisite in the clinical application of hepatic cryosurgery.

*© 2000 Cancer Research Campaign*

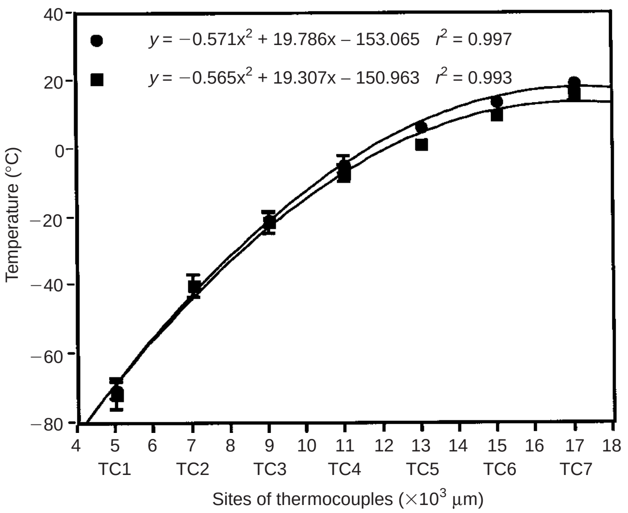

**Figure 5** Regression analysis between temperature at the end of the single (circles) and double freezing procedure (squares) and the sites of the seven thermocouples with respect to the centre of the cryoprobe, indicating significant ($P < 0.01$) correlations between both parameters. R, regression coefficient

### ACKNOWLEDGEMENTS

This study was supported by grants from the EU (BMH4-CT95-0875) and DFG (Me 900/3–1). BV is a recipient of a Heisenberg-Stipendium (Vo 450/6–1) from the DFG (Deutsche Forschungsgemeinschaft, Bonn-Bad Godesberg, Germany).

### REFERENCES

Adam R, Akpinar E, Johann M, Kunstlinger F, Majno P and Bismuth H (1997) Place of cryosurgery in the treatment of malignant liver tumors. *Ann Surg* **225**: 39–50

Bayjoo P (1992) Cryosurgery and the immune system. MD Thesis, University of Leeds

Bilchik AJ, Sarantou T, Wardlaw JC and Ramming KP (1997) Cryosurgery causes a profound reduction in tumor markers in hepatoma and noncolorectal hepatic metastases. *Am Surg* **63**: 796–800

Brown NJ, Bayoo P and Reed MWR (1993) Effect of cryosurgery on liver blood flow. *Br J Cancer* **68**: 10–12

Brown NJ, Pollock KJ, Bayoo P and Reed MWR (1994) The effect of cryotherapy on the cremaster muscle microcirculation in vivo. *Br J Cancer* **69**: 706–710

Charnley RM, Doran J and Morris DL (1989) Cryotherapy for liver metastases: a new approach. *Br J Surg* **76**: 1040–1041

Cozzi PJ, Englund R and Morris DL (1995) Cryotherapy treatment of patients with hepatic metastases from neuroendocrine tumor. *Cancer* **76**: 501–509

Dalton RR and Eisenberg BL (1993) Surgical management of recurrent liver tumors. *Semin Oncol* **20**: 493–505

Gage AA (1969) Cryosurgery for oral and pharyngeal carcinoma. *Am J Surg* **118**: 669–672

Hamad GG and Neifeld JP (1998) Biochemical, hematologic, and immunologic alterations following hepatic cryotherapy. *Semin Surg Oncol* **14**: 122–128

Jacob G, Kurzer MN and Fuller BJ (1985) An assessment of human cell viability after in vitro freezing. *Cryobiology* **22**: 417–426

Gill W, Fraser J and Carter DC (1968) Repeated freeze–thaw cycles in cryosurgery. *Nature* **219**: 410–413

Johnson LB, Krebs TL, Van Echo D, Plotkin JS, Njoku M, Wong JJ, Daly BD and Kuo PC (1997) Cytoablative therapy with combined resection and cryosurgery for limited bilobar hepatic colorectal metastases. *Am J Surg* **174**: 610–613

Lee FT, Mahvi DM, Chosy SG, Onik GM, Wong WS, Littrup PJ and Scanlan KA (1997) Hepatic cryosurgery with intraoperative US guidance. *Radiology* **202**: 624–632

Morris DL, Horton MDAC, Dilley AV, Walters A and Clingan PR (1993) Treatment of hepatic metastases by cryotherapy and regional cytotoxic perfusion. *Gut* **34**: 1156–1157

Neel HB, Ketcham AS and Hammond WG (1971) Ischaemia potentiating cryosurgery of primate liver. *Ann Surg* **174**: 309–318

Raab JM, Renaud LM, Brandt PA and Witt CW (1974) Effect of freezing on the microcirculation and capillary endothelium of the hamster cheek pouch. *Cryobiology* **11**: 508–518

Ravikumar TS and Steele G (1989) Hepatic cryosurgery. *Surg Clin North Am* **69**: 433–440

Ravikumar TS, Kane R, Cady B, Jenkins RL, McDermott W, Onik G, Clouse M and Steele G Jr (1987) Hepatic cryosurgery with intraoperative ultrasound monitoring for metastatic colon carcinoma. *Arch Surg* **122**: 403–409

Ravikumar TS, Steele G, Kane R and King V (1991) Experimental and clinical observations on hepatic cryosurgery for colorectal metastases. *Cancer Res* **52**: 6323–6327

Rubinsky B, Lee CY, Bastacky J and Onik G (1990) The process of freezing and the mechanism of damage during hepatic cryosurgery. *Cryobiology* **27**: 85–97

Schüder G, Vollmar B, Richter S, Pistorius G, Fehringer M, Feifel G and Menger MD (1999) Epi-illumination fluorescent light microscopy for the in vivo study of rat hepatic microvascular response to cryothermia. *Hepatology* **29**: 801–808

Seifert JK and Morris DL (1999) Indicators of recurrence following cryotherapy for hepatic metastases from colorectal cancer. *Br J Surg* **86**: 234–240

Smith JJ, Fraser J and MacIver AG (1978) Ultrastructure after cryosurgery of rat liver. *Cryobiology* **15**: 426–432

Steele G (1994) Cryoablation in hepatic surgery. *Semin Liv Dis* **14**: 120–125

Tatsutani K, Rubinsky B, Onik G and Dahiya R (1996) Effect of thermal variables on frozen human primary prostatic adenocarcinoma cells. *Urology* **48**: 441–447

Vollmar B, Glasz J, Leiderer R, Post S and Menger MD (1994) Hepatic microcirculatory perfusion failure is a determinant of liver dysfunction in warm ischemia-reperfusion. *Am J Pathol* **145**: 1421–1431

Vollmar B, Messner S, Wanner G, Hartung T and Menger MD (1997) Immunomodulatory action of G-CSF in a rat model of endotoxin-induced liver injury: an intravital microscopic analysis of Kupffer cell and leukocyte response. *J Leukoc Biol* **62**: 710–718

Weber SM, Lee FT, Chinn DO, Warner T, Chosy SG and Mahvi DM (1997) Perivascular and intralesional tissue necrosis after hepatic cryoablation: results in a porcine model. *Surgery* **122**: 742–747

Whittaker DK (1984) Mechanisms for tissue destruction following cryosurgery. *Ann R Coll Surg Engl* **66**: 313–318

Zhou XD, Tang ZY, Yu YQ, Weng JM, Ma ZC, Zhang BH and Zheng YX (1993) The role of cryosurgery in the treatment of hepatic cancer: a report of 113 cases. *J Cancer Res Clin Oncol* **120**: 100–102

*© 2000 Cancer Research Campaign*
